# Breaking the chains: exploring gender inequality in Türkiye through the eyes of women NGO members

**DOI:** 10.3389/fsoc.2024.1491058

**Published:** 2024-11-01

**Authors:** Gokhan Savas, Dilek Çakır

**Affiliations:** ^1^College of Arts and Sciences Department of International Studies, American University of Sharjah, Sharjah, United Arab Emirates; ^2^Political Science and Public Administration Department, Social Sciences University of Ankara, Altındağ, Türkiye

**Keywords:** gender attitudes, civil society, gender (in)equality, women rights, patriarchy

## Abstract

In Türkiye, women’s NGOs have gained significant influence in gender politics, especially since the country’s turn towards neoliberalism. A survey conducted among 735 members of women’s NGOs revealed that, contrary to expectations, many members hold gender inequitable attitudes, highlighting a lack of gender consciousness within these organizations. Key findings indicate that support for gender equality is higher among participants in Ankara than in Istanbul, and that factors such as education, political ideology, and socio-economic status significantly shape these attitudes. The persistence of patriarchal beliefs within these organizations suggests the need for a deeper analysis of the socio-political and structural barriers that hinder gender equality. This study provides critical insights into the intersection of civil society, gender attitudes, and advocacy in Türkiye.

## 1 Introduction

Civil society has become as a major focus of attention in academic literature and public discourse. Women civil society organizations, often regarded as vehicles for gender equality and empowerment, play a significant role in shaping gender politics. However, civil society does not inherently foster progressive change, as Howell and Mulligan (2004) point out, civil society can reinforce patriarchal ideologies and conservative beliefs. This paper draws on Bourdieu’s concept of symbolic violence to explore how patriarchal domination persists within women’s NGOs. Symbolic violence refers to the subtle ways in which domination is maintained through internalized social hierarchies, even among groups advocating for equality. Additionally, Gramsci’s theory of hegemony provides a lens for understanding how patriarchal values become internalized within civil society, shaping the beliefs and actions of NGO members. This theoretical perspective allows for a critical examination of how NGOs, despite their advocacy for women’s rights, may inadvertently sustain gender inequitable attitudes. By applying these theoretical lenses, this paper aims to contribute to the academic discourse on gender and civil society by challenging essentialist views of women’s organizations as inherently feminist. The findings from the empirical analysis of women’s NGOs in Türkiye will provide valuable insights into the complex dynamics of patriarchy within civil society and the broader implications for gender politics and advocacyWomen’s NGOs in Türkiye operate within a complex socio-political environment that has become increasingly hostile to progressive movements. This study explores the challenges faced by these organizations in advancing gender equality, particularly in light of the rise of patriarchal and conservative ideologies. However, this issue is not unique to Türkiye. Globally, there is a growing backlash against gender equality and feminist movements, fueled by anti-woke, misogynist, and incel ideologies. These ideologies, often amplified by social media and political rhetoric, have created a climate where the effectiveness and impact of women’s NGOs are being diminished. Recent research has highlighted these global trends (Fitzsimmons et al., 2023; Özbilgin and Erbil, 2024a; Özbilgin and Erbil, 2024b).

In many countries, including Türkiye, this backlash is contributing to the entrenchment of patriarchal norms and the resistance to gender equality. Women’s NGOs are finding it increasingly difficult to challenge these ideologies, as they navigate a landscape that is often unsupportive, if not outright hostile, to their mission. By situating this study within this broader global context, we highlight the significance of examining the internal dynamics of women’s NGOs, particularly how they may both challenge and inadvertently reinforce the patriarchal structures they seek to dismantle.

Understanding the challenges women’s NGOs face in Türkiye provides valuable insights for organizations operating in similarly challenging environments around the world. This research not only contributes to the academic discourse on gender and civil society but also offers practical insights that can inform strategies for enhancing the effectiveness of women’s NGOs in the face of growing socio-political opposition.

After Türkiye’s neo-liberal turn in the 1980s, women’s civil society organizations have proliferated and diversified. Since then, these organizations play an influential role in promoting women’s political participation and representation especially through their ability to mobilize and organize women, influencing public opinion and engaging with state and other key stakeholders.

To critically assess the complex dynamics within women’s NGOs, this paper draws on Bourdieu’s concept of symbolic violence and Gramsci’s theory of hegemony. Bourdieu’s framework helps to understand how patriarchal domination is subtly maintained within civil society organizations, often through internalized social hierarchies that perpetuate inequitable gender attitudes. Meanwhile, Gramsci’s theory of hegemony provides a lens for understanding how dominant ideologies, such as patriarchal values, become internalized even among groups advocating for equality. These frameworks guide the analysis of the data, allowing for a nuanced exploration of how women’s NGOs in Türkiye can simultaneously challenge and reinforce gender inequality. By using these frameworks, the study seeks to contribute to the academic discourse on gender and civil society by challenging the assumption that women’s NGOs inherently promote feminist ideals. This theoretical approach enables a deeper analysis of the persistence of patriarchal values within advocacy organizations and offers insights into how gender inequality can be sustained through internalized social hierarchies. The findings of this study will be interpreted through this lens, offering new perspectives on how civil society can simultaneously advance and hinder gender equality.

This study contributes both theoretically and practically to the discourse on gender inequality and civil society. Theoretically, it challenges essentialist assumptions that women’s organizations inherently promote feminist principles and gender equity. By examining gender attitudes within women’s NGOs, this paper highlights the complexity of civil society as both a site for empowerment and a potential space for reinforcing patriarchal norms. Practically, the findings provide insights that can inform policy-making and organizational practices within women’s NGOs, helping to enhance gender consciousness and activism. This research is especially relevant for understanding the persistent barriers to gender equality within seemingly progressive spaces. To this end, this paper seeks to answer the following research question: *To what extent do the members of women’s NGOs in Türkiye hold gender-equitable attitudes, and how do these attitudes intersect with socio-political and demographic factors such as age, marital status, and political ideology?* This research is grounded in the broader context of Türkiye’s neoliberal turn and its influence on civil society and women’s movements. Within this context, a survey was carried out as a part of a broader initiative known as “Evaluating Women’s Policies from the Perspective of Women’s Organizations” that received funding from Scientific Research Coordination Unit of the Social Sciences University of Ankara. The quantitative data reveals that women, on contrary to the expectations, members of women NGOs hold gender inequitable attitudes. To understand this unexpected result, it is primarily important to escape from essentialism and accepts that all women organizations do not have to adhere feminist principles. Additionally, there are specific structural constraints in Türkiye that restrict the power and impact of women’s NGOs. Thus, this data should be enriched with the history of women NGOs and women rights in Türkiye. In other words, it is necessary to take into account theoretical debates and historical, political and legal specificities that has conditioned the intricate relations among civil society and state authority towards gender equality so that we can gain a deeper understanding of complex dynamics at play.

Despite the growth and variety of women’s associations in Türkiye, patriarchal attitudes remain entrenched and hinder their ability to effectively challenge gender-based hierarchies and promote gender awareness. To assess if patriarchy persists within women organizations, this paper examines the gender attitudes of women NGO members. The persistence of gender inequitable attitudes cannot be explained exclusively by personal factors. It is essential to first provide a thorough examination of the state of women’s rights and the role of women’s NGOs in Türkiye. This context allows for a deeper understanding of the quantitative data, which analyzes how demographic factors such as age, marital status, socio-economic situation, and political ideology influence gender attitudes among NGO members.

Since the withdrawal from the Istanbul Convention in 2021, Türkiye has experienced a resurgence of traditionalist ideologies promoting family values, while anti-LGBT discourse has become increasingly prevalent. This has created a challenging socio-political environment for women’s rights and gender equality. The shift in the state’s position has had a significant impact on the landscape in which women’s NGOs operate. These developments provide a critical backdrop to understanding the persistence of patriarchal norms within civil society, even as these organizations advocate for gender equity. This context is essential to analyzing the evolving dynamics of gender inequality in Türkiye and understanding the contemporary challenges women’s NGOs face.

This study builds on existing debates surrounding the role of civil society in promoting democratic values and gender equality, particularly within the Turkish context (Howell and Mulligan, 2004). By focusing on women’s NGOs, the research extends prior work on gender attitudes (Öztop and Finkel, 2015) and challenges the assumption that civil society organizations are inherently progressive. Moreover, this study addresses gaps in the literature on how patriarchal structures can persist even within organizations that advocate for women’s rights.

This article aims to provide a thorough understanding of the issue by first emphasizing the need to avoid assuming that women’s NGOs inherently hold feminist principles. It then offers a brief history of the development of women’s rights and NGOs, in order to explore the complex relationships between the state, women NGOs and women’s rights. Despite the increase in the number and diversity, they still have limited feminist capacity, which might condition gender inequitable attitudes among their members. After that, the article examines demographic variables through the use of survey results. The analysis illustrates how gender intersects with other social categories, such as age, socio-economic status, and marital status, to influence gender perception.

## 2 Defining civil society: challenging essentialist presumptions

A wide range of intellectual and political discussion on the concept of civil society has risen. However, the general understanding of civil society is based on liberal theory (Osborne, 2021, p. 175), which views civil society as the area outside of the state where here all relations of individuals are performed (Frankel, 1983, p. 26). This is considered a space where people can voluntarily come together to express their opinions and negotiate their interests collectively (Narayan-Parker, 2005, p. 342). According to Keyman, civil society is the area of public deliberation where the relationship between the state and society/individuals is settled (Keyman, 2000). Eventually, it is seen as a mechanism of balance between individual interests and central state (Ayhan, 2020, p. 47). Habermas, a key figure in the public sphere debates, argues that civil society [voluntary & external to state] plays an important role in forming public opinion and strengthening democratic practices (Habermas, 1997, pp. 50–54). The fundamental principle of this liberal view is the emphasis on individual rights and freedoms. In this regard, civil society aims to protect individuals from state tyranny and ensure that all individuals have equal rights and liberties (Jailobaeva, 2007, p. 3; Tocqueville, 2016). In practice, civil society encompasses a wide range of non-governmental organizations that are voluntary and vary by size, structure, and platform, ranging from local to international organizations (VanDyck, 2017). According to this view, civil society has a political role, which involves promoting individual rights and freedoms and strengthening the public sphere to prevent state tyranny. As Schechter (2016) suggests, civil society now plays a crucial role in the possible democratization of the world.

However, civil society does not inherently possess the potential to promote pluralism and democracy. According to Howell and Mulligan (2004), civil society can have both positive and negative effects on democracy. Despite the common belief that civil society has the potential to promote democracy and liberalism, it is not regulated and thus can be vulnerable to anti-democratic, intolerant, ultra-nationalist, and patriarchal ideologies. In terms of women civil society organization which are typically founded and run by women to advocate for women’s rights (UN Women, 2020; Esim and Cindoğlu, 1999), their aim is often assumed to address the needs and priorities of women in the public sphere, promoting gender equality and empowering women. However, it is not accurate to assume that these organizations inherently promote gender equality. Some of these organizations may not aim for these objectives, and they may even reinforce patriarchal and anti-democratic beliefs. As well demonstrated by Sombatpoonsiri (2020) civil society which is a space for various groups and ideologies to express their interests and beliefs, might be abused by fundamentalists to denounce democracy and strengthen anti-democratic values. It has the potential to become a platform where fundamentalist groups could practice their identity politics and anti-democratic understanding (Keyman and İçduygu, 2003, p. 221). Bourdieu’s concept of symbolic violence is particularly useful in understanding how patriarchal domination is maintained within women’s NGOs. Symbolic violence refers to the subtle and often unconscious reinforcement of social hierarchies, which can explain why gender inequitable attitudes persist even within organizations that advocate for gender equality. Similarly, Gramsci’s theory of hegemony suggests that dominant ideologies, such as patriarchal values, can become internalized within civil society, shaping the actions and beliefs of groups advocating for change. Alternatively, agency theory can be used to explore how women within these NGOs navigate and resist patriarchal constraints while attempting to promote gender equality.

In Turkish case, it is important to have a nuanced understanding of the complex relation among the advancement of women’s rights, the role of women’s NGOs, and their interaction with the state. By examining these intricate relations, the following discussion will offer a contextual structure for comprehending the survey results, which reveal that members of women’s NGOs hold gender inequitable attitudes.

## 3 Constrains on the influence and impact of women NGOs in Türkiye

The advancement of women’s rights cannot be attributed exclusively to the rise of feminist ideology and women’s NGOs. While Türkiye has achieved notable progress in women’s rights, there is still more to be done. To understand the impact of women’s NGOs on gender politics and women’s rights, it is necessary to examine their role despite the fact that these organizations may not consistently adhere to feminist principles or promote gender equality. Without understanding these contextual nuances, including the surrounding gender structure and political context, any quantitative data on the gender perceptions of individual NGO members would not be sufficient to grasp gender inequality in Türkiye. To this end, (1) the strong state tradition that curbs the influence of women NGO’s (2) the interplay between gender perceptions, the emergence of women’s NGOs, and the development of women’s rights since the 1980s will be examined, accordingly.

### 3.1 State authority limiting agency of women NGOs

Until 20^th^ century, women in Turkish society had limited roles centered around being mothers and homemakers which result in restricted participation on political and social spheres. In fact, the early attempts for the emancipation of women began in Türkiye during the early 1800s, particularly after the Tanzimat era (Abadan-Unat, 1978, p. 291; Konan, 2011). Despite certain progress during Ottoman Constitutional Era (Abadan-Unat, 1978, p. 293; Konan, 2011), Turkish Republic, adopting a radical secularization and Westernization project, has broken any ties from the formerly Sharia law and eliminated religious foundations including the Caliphate and Ministries of Sharia. This secularization project has liberated the women’s lives hemmed in by religious restrictions. The historical struggle for women’s political rights in Türkiye, led by figures like Nezihe Muhiddin and organizations such as the Türk Kadınlar Birliği and Kadınlar Halk Fırkası, laid the foundation for Türkiye’s progressive stance on women’s political participation in the early 20th century. These movements played a crucial role in securing women’s suffrage and positioning Türkiye as a leader in gender equality. However, despite these early gains, the current state of women’s NGOs suggests a reversal or stagnation in gender equity progress. Acknowledging these historical contributions enriches our understanding of the current challenges faced by women’s NGOs as they continue to advocate for gender equality in a changing socio-political environment.

The process of secularization in Türkiye had significant implications for women’s lives. During the Early Republic, there were significant advancements towards legal emancipation of women (Kaymaz, 2010, p. 333). Turkish Civil Code (1926) prioritized gender equality, ensuring that women were not subjected to any discrimination in their personal status within marriage, family, or public life. The Civil Code prohibited forced marriages, child marriages, polygamy, and Islamic marriages without an official civil marriage. Additionally, the Civil Code granted both partners equal rights to divorce and child custody. Another significant reform during the Early Republican period was the law on the unification of education in 1924 (*Tevhid-i Tedrisat Kanunu*), which provided women with the right to education by centralizing all educational institutions under the Ministry of Education and banning previous religious schools (Turan, 2000). The Turkish constitution provides both men and women with the right to public education that is compulsory and free of charge (Aydin, 2015). Women are also granted political rights, including the right to vote and be elected, under general suffrage (Aydin, 2015, p. 94; Gökçi̇men, 2008, p. 22). These measures have helped to achieve greater gender equality in Türkiye. However, as Tepe points out, these rights are seen as a gift from the Republic and women is portrayed as grateful beneficiaries. She therefore describes the situation of women during Early Republic as emancipated but unliberated. Although legal emancipation is a necessary step towards liberation, it does not guarantee complete liberation (Tepe, 2014). During Early Republican Period in Türkiye, women’s rights were conceived as part of Republic’s political modernization project. Women’s legal rights during this period were not viewed through a gender perspective, and women did not have a say in the entitlement of women’s rights.

The prevalence of strong state tradition obstructed the flourishment of women civil society organizations until 1960’s. The state justified its control over civil society by arguing that it had already taken the lead in promoting women’s rights even before there were any demands from women themselves. Therefore, there was no need for women to organize (Kardam and Ertürk, 1999). In Türkiye, the women’s movements have risen particularly after the neo-liberal turn in 1980s as it led to the formation of numerous women NGOs. Since then, these organizations have become more diverse and advanced, which result in significant developments in terms of women rights. By engaging in dialogue with the state, women NGOs have succeeded in representing women as a social group and have compelled the state to incorporate women’s interests into organizational and political programs, procedures, projects, and goals (Kardam and Ertürk, 1999). As a result, gender accountability has increased significantly, and Türkiye has made notable legal, institutional, and political progress towards gender equality. The progress made so far in terms of gender equality is significant, but it is not yet satisfactory, as will be discussed next.

### 3.2 Progress and remaining challenges: the rise of women NGOs and the evolution of Women’s rights

With the transition to economic liberalization especially after 1990’s, Turkish government gave priority EU enlargement process which has immense impacts on socio-political agenda. This process has immense influence on the proliferation of women rights together with the broadening influence of women NGO’s (The New Global Order, 2022). Following the prospect of EU accession, Türkiye has committed itself to democratization and pluralism (Seçkinelgin, 2016, p. 749). Many civil society organizations have emerged as a new autonomous societal space where different groups represent and articulate their interest (Keyman and İçduygu, 2003, p. 221; Göle, 1994). Within this picture, women NGOs have considerably expanded their influence and activities. Since ensuring women equality and women rights is indispensable in terms of the consolidation of democracy, European integration process has further enforced the government to collaborate with women NGOs for proliferation of women rights. The cooperation with the European women NGOs (Eslen-Ziya and Kazanoğlu, 2020; Tekeli, 1992) has further contributed for the establishment of gender equality.

Considerable progress has been made in Türkiye regarding legal and institutional practices aimed at achieving gender equality. This is evident in the amendments made to the Turkish Constitution (art. 10, 41, and 66), which included affirmative action measures and sought to ensure that women had equal constitutional status. Furthermore, The Criminal Law removed any traces of patriarchal gender hierarchy by explicitly stating that “no discrimination shall be made between persons with respect of sex.” Similarly, the 2001 Turkish Civil Code introduced legal measures to guarantee gender equality within families (Marin, 2015). Türkiye not only implemented legal reforms but also prioritized the Convention on the Elimination of All Forms of Discrimination against Women (CEDAW) in its gender policies, giving it precedence over national laws (European Parliement, 2012). Additionally, Türkiye made significant institutional changes to guarantee equal access to education, training, healthcare, and job opportunities for everyone. To this end, the General Directorate on the Status of Women—which is organized under the Ministry of Family, Labor and Social Services—developed a National Action Plan for Societal Gender Equality for the period 2008–2013, which focus on key priority areas such as education, economy, poverty, power and decision-making, health, media, and the environment with the goal of eliminating gender-based inequalities. The Turkish Government’s 9th Development Plan also reinforces declared the importance of gender equality by emphasizing the necessity of increasing women’s labor force participation. The General Directorate on the Status of Women in Türkiye also implemented the National Action Plan for Societal Gender Equality for 2008–2013, which was created as part of the EU’s Twinning Project (2008) in order to promote gender equality and strengthen institutional capacity. The Action Plan targets seven specific areas namely (1) education, (2) economy, (3) poverty, (4) power and decision-making, (5) health, (6) media, and (7) environment (Savaş, 2018).

Despite efforts to address gender inequality in Türkiye, the country’s situation remains unsatisfactory. The World Economic Forum’s 2020 Gender Gap Index ranks Türkiye as 133rd out of 156 countries (UNFPA, 2020), while the Gender Inequality Index (GII) places it at 66th out of 162 countries (UN, 2022). The European Parliament’s report on Gender Equality in Türkiye highlights that, between 2000 and 2019, although both the Human Development Index (HDI) and the Gender Development Index (GDI) increased, the GDI did not increase as quickly as the HDI (UNDP, 2022b, p. 3). The HDI measures average achievement in terms of three indicators: (1) long and healthy life including access to health services, (2) knowledge including access to education and (3) a decent standard of living including income (UNDP, 2022a). The GDI measures gender gaps in human development achievements using these same three indicators. The report notes that the slower increase in GDI indicates that progress in addressing gender gaps has slowed (UNDP, 2022b). Although there has been some advancement in terms of gender accountability of women’s NGOs and progress in women’s rights, Türkiye is still far away from achieving gender equality as patriarchal norms continue to be deeply rooted, which will be further elaborated on.

## 4 Persistence of patriarchy and gender (in)equality perception

Patriarchy designates women with subordinate positions in society, often attributing them emotional and passive characteristics, while men are expected to possess rationality, activeness, and superiority. Women’s identities are often defined solely by their roles as wives and mothers, rather than as individuals with their own desires and aspirations. Patriarchy is a pervasive force that is deeply rooted in social relationships and present in all spheres of social life, including politics, economics, social structures, and religion. It leads to gender inequalities by constraining women’s access to education, employment, political participation, and healthcare (Joseph, 1996, p. 14). Despite significant progress made in women’s rights after the 1990s, patriarchy remains a pervasive force in Türkiye, limiting gender equality and perpetuating gender-based discrimination. Statistics clearly demonstrate the existence of gender disparities across social, economic, and political domains. In 2019, 20.8% of the total population aged 25 and over were university graduates. When this was analyzed by sex; only 18.5% of females and 23.1% of males were university graduates. In terms of female employment, it was less than half of male’s employment rate. In 2019, the proportion of those 15 and over who were employed was 45.7% in Türkiye. Out of this proportion, only 28.7% were females, while 63.1% were males. According to the results of the household labor force survey; in 2019, the proportion of those who were 15 years of age and over and in employment was 45.7% in Türkiye. To give an example of gender disparities in political sphere, the percentage of female deputies in parliament was 17.3%, with 101 female deputies and 483 male deputies out of a total of 584 deputies in 2020 (TUIK, 2021; Table 1).

**Table 1:** Selected indicators by sex.

Selected indicators by sex, 2019	Total	Male	Female
The proportion of illiterate population (25+ age)	4.1	1.2	6.9
The proportion of higher education graduates (25+ age)	20.8	23.1	18.5
Employment rate (15+ age)	45.7	63.1	28.7
Labor force participation (15+ age)	53.0	72.0	34.4
Unemployment rate (15+ age)	13.7	12.4	16.5

The table was created by using TUIK data. From TUIK (2021) by https://data.tuik.gov.tr/Bulten/Index?p=37221&dil=2.

Gender perception of women NGO members bear signification as they play a crucial role in gender politics. This perception can affect their effectiveness and ability to contribute to gender (in)equality. By identifying and addressing the determining factors, it is possible to change and challenge it in order for a greater gender equality and inclusion. This can also inform the development of policies and interventions by stakeholders to eliminate gender inequality, which might further the feminist agenda.

### 4.1 Survey design: measuring gender perception

A survey was carried out as a part of a broader initiative known as “Evaluating Women’s Policies from the Perspective of Women’s Organizations” funded by Scientific Research Coordination Unit of the Social Sciences University of Ankara. It was designed as a quantitative survey research involved 735 participants who were members of women’s associations in Istanbul and Ankara. The survey was conducted from November 2017 to February 2018. The decision to use a quantitative survey approach was driven by the need to collect data from a large and diverse group of women NGO members in order to assess their gender attitudes comprehensively. A quantitative method is best suited for this study because it allows for the measurement of attitudes across different demographic variables such as age, education, and political ideology, providing a more objective and generalizable understanding of the factors influencing gender inequality perceptions. By employing standardized survey questions, this method ensures consistency and comparability across respondents, making it an ideal approach to exploring the complex dynamics of gender attitudes within women’s NGOs.

As a part of this project, we first updated the list of women’s associations in Istanbul and in Ankara as of November 2017. In doing that we used the official web page of the Ministry of Internal Affairs, Associations Desk online system of associations. We downloaded all the associations and classified women’s associations using a 3-step procedure. Firstly, we included all the associations that stated that their operations were related to “women’s rights.” Secondly, we further searched for associations using feminine definition words such as “women,” “girl,” “lady,” “mother,” etc. Lastly, we omitted the subsidiary branches of associations and retained only the main quarters of the associations. After this selection procedure, we ended up with a total of 292 associations, 180 from Istanbul and 112 from Ankara. In order to reach 5 respondents from each association for diverse perspectives, we aimed to reach 1,050 respondents in total at the 5% confidence interval. Unfortunately, we were unable to reach 1,050 respondents due to low response rates particularly in Istanbul. Thus, the final sample remained confident with 9% confidence interval in Istanbul and %7 confidence interval in Ankara, consisting of 365 and 370 respondents from these cities, respectively. At the end, we had data from 735 women from 151 different women NGOs (Table 2).

**Table 2:** Sampling method and confidence levels.

City	Sample Aim (5% confidence interval) (%95 confidence level)	Final sample
Ankara	87 NGOs × 5 members = 435	76NGOs × (max. 5 members) = 370 (7% Confidence interval) (%95 Confidence level)
İstanbul	123 NGOs × 5 members = 615	75NGOs × (max. 5 members) = 365 (9% Confidence interval) (%95 confidence level)
Total	210 NGOs = 1,050	151 NGOs = 735

#### 4.1.1 Dependent variable

Gender inequality starts with individual’s understanding and awareness of socially expected categories of gender roles (Öztop and Finkel, 2015), and it has several dimensions including family life, education, economics, and social life. Having 10 statements about each different dimension this study has measured perceptions towards gender inequality. We have created the index of gender inequality by including 10 items for 10 different dimensions of gender equality. The respondents were expected to rate the level of agreement between 0 not agree at all to 10 completely agree to the following items;

- Since men are responsible for family livelihood, men should be given more salary than women.
- Women are more suited to social sciences; men are more prone to numerical sciences.
- Because women are more emotional than men, they have difficulty in doing professions such as management.
- Men are more prone to politics than women.
- Man should decide how to use the income in the family.
- The level of education among men should always be higher than that of women.
- The main duty of the woman in society is motherhood.
- Girls and boys should benefit equally from the financial means of the family.
- A man should not be expected to be involved in housework such as ironing, laundry, child care.
- A woman who is unhappy in her marriage must still maintain her marriage.

The index is quite reliable given that its Cronbach’s alpha is 0.84.

#### 4.1.2 Independent variables

We have created an index of political participation by expecting them to choose either they agree or not agree with the following 10 statements:

- Political issues take my attention.
- I follow the news and the agenda.
- I participate in political meetings, rallies and/or marches.
- I follow the political debate programs on TV.
- I vote in elections.
- I follow the debates in the parliament.
- I volunteer activities for election campaigns.
- I am a member of a political party.
- I like to chat in political matters.
- I read books on political issues.

The index is reliable given that its Cronbach’s alpha is 0.75.

We asked the following question in order to measure self-evaluation of religiosity (rating between 1-not religious at all to 11-very religious);

*Where would you see yourself if you would evaluate your religiousness in terms of fulfilling religious practices?*

We measured whether the respondents were following the policies that are developed on women’s issues by asking the question that;

Can you tell to what extent do you follow the developments on women’s policies in last 15 years? (Never, Rarely, Sometimes, Often, Always)

We have included the control variables of personal monthly income, marriage, age, education, city, whether respondents have administrative duty in association, another NGO membership, political ideology, and religiosity. As such, the goal is to define the factors that affect women’s gender inequality perceptions.

When selecting control variables, we try to consider the relevant factors that can influence individual’s gender inequality perception. By examining the pertinent literature, we try to identify the most valuable variables to consider. Demographic variables such as age, gender and marital status is considered to be important factors affecting gender perception. It was hypothesized that women with higher levels of education and job status would exhibit greater awareness of gender issues because they have overcome gender inequality barriers. Marital status is also seen as a significant factor (Kane and Sanchez, 1994) as marriage often involves having children and raising family, which might perpetuate traditional gender roles. Accordingly, age is considered to be a determining factor and the increasing prevalence of feminist ideology among young people led us to expect a greater level of gender sensitivity among younger group (Kehn and Ruthig, 2013). NGO positions and membership were also included as potential factors affecting gender perception, given their relationship with activism among women. Then, religious affiliation, particularly with Islam, was considered due to its historical association with defending traditional gender roles (Campbell et al., 2018). Lastly, political ideology was included as a potential factor, as conservative ideologies tend to support patriarchal structures while the new left emphasizes women’s rights, democracy, and human rights. The inclusion of these control variables allowed us to uncover the determinants of gender perception.

If a woman chooses to join a women’s NGO, she is often assumed to be motivated by a desire to promote to gender equality and women empowerment. We might expect that members of such organizations would be highly aware of gender issues and show little gender inequality in their attitudes. Yet, the respondents’ actual attitudes towards gender equality are not as gender-conscious as expected, and these gender inequitable attitudes vary depending on demographic variables and personal traits such as political ideology, age, resident city, and level of religiosity.

To contextualize the findings, it is important to understand the significance of Istanbul and Ankara, the two cities where the study was conducted. Istanbul, with a population of approximately 20 million, is the largest city in Türkiye and serves as the country’s cultural and economic center. It is a highly diverse metropolis shaped by internal migration, making it a hub for both modernity and traditional values. Similarly, Ankara, the nation’s capital and second-largest city, is home to key government institutions, including the Parliament and major ministries. While also a destination for internal migration, Ankara’s political and bureaucratic focus gives it a unique socio-political landscape distinct from Istanbul’s. Both cities play pivotal roles in shaping Türkiye’s political discourse and are central to understanding the broader dynamics of gender inequality within the country.

### 4.2 Survey results

This chapter discusses the survey results. After presenting the descriptive statistics, gender (in)equality perception and its determining factors are discussed in detail.

#### 4.2.1 Descriptive statistics

Respondents in this study are equally scattered for Istanbul and Ankara. 50.34% of them are from Ankara, and 49.66% come from Istanbul. 52.52% of respondents have administrative duty in their association, and the majority of them (94.15%) do not have any other women’s association membership. Based on descriptive results, it can be said that women in this research seem to be interested in current politics. Only 1.77% of them never follow politics, and 9.80% of them rarely follow politics. Many of them report that they follow politics sometimes (33.06%), often (34.97%) and always (20.41%). This result consists with the political participation index. According to the index, the mean score is 12.571 out of 18. Overall, women in this research are interested in politics (Table 3).

**Table 3:** Descriptive statistics.

Variable	F	%
Administrative duty in association		
Yes	386	52.52
No	349	47.48
Another women association membership		
Yes	43	5.85
No	692	94.15
Income		
0–1,500 TL	147	20.00
1,501–3,000 TL	258	35.10
3,001–4,500 TL	120	16.33
4,501–6,000 TL	39	5.31
6,001–7,500 TL	16	2.18
7,501 and above	8	1.09
Marital status		
Married	299	40.68
Single	436	59.32
Political ideology		
Leftist	380	51.70
Rightist	241	32.79
None	114	15.51
Region		
Ankara	370	50.34
Istanbul	365	49.66

	Mean	St. Dev.	Min.	Max.
Political Participation Index	0.40	0.24	0	1
Age	41.322	12.736	19	78
Education	6.002	2.053	1	9
Gender (In)equality Index	0.27	0.21	0	1
Religiosity	0.56	2.617	0	1

Given that the poverty line in Türkiye was about 5,000 Turkish liras (TL) as of 2018, the majority of women lived under poverty line. 20% of them indicate that their income is in between 0 and 1,500TL, for 35.10% of them it is 1,501–3,000TL. Also, in Türkiye people usually hesitate to talk about their income so that 20% of the respondents in this study do not want to report their income. While the income data presented in this study provides valuable insights into the socio-economic status of respondents at the time of the survey, it is important to interpret these findings cautiously due to the significant economic changes Türkiye has experienced since 2017. The country has faced considerable economic instability, including periods of hyperinflation, currency devaluation, and rising living costs, which have drastically altered income distribution and economic realities. As a result, the income categories used in this survey may no longer accurately reflect the current socio-economic status of respondents or the broader population. These economic shifts are likely to have profound implications for gender inequality, as financial insecurity often exacerbates existing disparities. Future research should account for these changes to provide a more up-to-date analysis of the relationship between income and gender perceptions in Türkiye. Nevertheless, the data remains useful for understanding the socio-economic landscape of women’s NGO members at the time the survey was conducted, but with the understanding that the economic conditions have since shifted.

Among women’s NGO members, while 59.32% of them are single, 40.68% of them are married. When we look at the political ideology of respondents, we can say that we have more women who identify themselves as leftist rather than rightist. Among the respondents, 15.51% did not identify with a specific political ideology and selected ‘none.’ While this might indicate apoliticism, it is crucial to consider the possibility that respondents were hesitant to disclose their political views due to privacy concerns or social pressures. Given the political climate in Türkiye, some individuals may refrain from revealing their affiliations out of fear of potential repercussions or stigma. Thus, the ‘none’ category may not reflect genuine disengagement from politics but could instead represent a strategy to avoid being labeled or categorized. This possibility should be kept in mind when interpreting the data, as it adds complexity to understanding the political orientations of the respondents.

While the average age of respondents is 41.32, the average level of education is around 14 years of formal education. This suggests that educated women are more likely to challenge patriarchal norms, consistent with prior studies (Anyanwu, 2016; Öztop and Finkel, 2015). However, it is essential to ask why education plays such a critical role. Education not only provides knowledge but also exposes women to feminist ideologies and gender-sensitive policies, which may explain the more progressive gender attitudes observed. This finding also underscores the importance of integrating gender studies into educational curricula to foster gender consciousness at an institutional level.

The respondents were asked how they would see themselves if they would evaluate their religiousness in terms of fulfilling religious practices, and after normalized between 0 and 1, the average score is 0.56. According to the normalized index scores of political participations, the average is 0.40.

#### 4.2.2 Gender inequality index and determining factors

The study has Gender Inequality Index and it has scores between 10 (lowest inequality) and 86 (highest inequality). After normalized between 0 and 1, the average index score of the participants is 0.27 (Table 4).

**Table 4:** OLS regression on gender inequality index.

Variable	β	SE
Age	−0.001	0.001
Ankara	0.006	0.019
Education	−0.009***	0.004
Income	−0.009	0.008
Single	0.010	0.017
Rightist	0.097***	0.021
None	0.024	0.024
Administrative duty	−0.028	0.019
Another membership	0.006	0.035
Political participation	0.094**	0.006
Religiosity	0.194***	0.035
R-squared	0.23	
*N*	735	

Women do not have another women’s association membership; do not have administrative duty in association; are married; from Istanbul; and who are leftist are reference categories. β denotes for unstandardized coefficients. *p < 0.05, **p < 0.01, ***p < 0.001.

We did OLS regression to see the factors affecting women’s gender inequality perceptions.

The data reveal that many members of women’s NGOs still hold gender inequitable attitudes, even in organizations committed to promoting gender equality. This can be interpreted through Bourdieu’s concept of symbolic violence, where dominant patriarchal structures are internalized, even unconsciously, by those advocating for change. These attitudes reflect the subtle ways in which societal hierarchies continue to be reproduced, suggesting that the internalization of patriarchal norms within NGOs may be preventing more transformative changes.

##### 4.2.2.1 Religion

Acknowledging that religion is a complex and fluid concept with diverse interpretations and practices that vary across different cultural and historical contexts, it is widely discussed in literature that religious beliefs and involvement tend to be negatively associated with perceiving gender equality (Seguino, 2011; Schnabel, 2016; Goldscheider et al., 2014). Islamic traditions and norms in particular often contribute to the continuation of gender disparities and the subjugation of women in society because Islamic discourse often embodies patriarchal values and ascribes secondary roles to women (Arat, 2010). Consequently, both Islamic belief and involvement can potentially act as explanatory factors that influence not only attitudes towards gender, but also the existence of disparities in various spheres such as social, economic, and political domains (Klingorová and Havlíček, 2015). Similarly, our findings indicate that a higher level of religious commitment is directly linked to holding more gender-inequitable attitudes, with a one-point increase in religiosity index corresponding to a 1.75-point increase in inequality index score.

##### 4.2.2.2 Political ideology

Women’s political opinions significantly influence their gender roles perceptions. Respondents with right-wing ideology have a higher score on the gender inequality index than leftist women. Both contemporary and historical far right draws on gender as a meta-language and family motif as a propaganda rhetoric (Heinemann and Stern, 2022). The right-wing often uses the term “gender” to create moral panic around issues related to sexuality, gender identity, and reproduction. They consider gender as a harmful ideology that sexualizes children, threatens heterosexual families, and gives unfair advantages to women over men. The term “gender” has taken on a negative connotation, associated with moral decay, corruption, and left-wing extremism (Korolczuk and Boczkowska, 2022). (New) left, on the other hand, demonstrates greater awareness towards the exclusion of social groups and prioritizes women’s rights. The left parties often maintain closer connection with the feminist movement and promote empowerment of women (Keith and Verge, 2018). Consequently, they tend to implement gender quotas to address the power imbalances (Freidenvall, 2013; Kittilson, 2011).

##### 4.2.2.3 Age

Age is another factor that has a negative correlation with gender equality. With Türkiye ‘s shift towards neoliberalism, gender politics has become more significant, and feminist ideology has gained power through civil society after 1980’s. Women’s civil society organizations have been established independently and in opposition to the state (Arat, 1994). Although progress is still insufficient, women’s agency and voices have become more important and have led to significant changes in challenging institutionalized gender disparities. As a result, especially since the 1990s, the advancements in gender issues have influenced individuals’ personal perceptions of gender, and younger generations brought up in this environment tend to be less tolerant of gender inequalities. Similarly, our research shows that for each year increase in age, the inequality index score goes up by 0.088, indicating that older individuals tend to hold more gender-inequitable attitudes.

##### 4.2.2.4 Resident city

Respondents living in Ankara significantly have more gender equal perceptions compared to those living in Istanbul. This difference could be explained by the specific position of Ankara as the capital of Türkiye. Not only the political parties but also numerous government agencies, non-governmental organizations and grass-root organizations has centered in Ankara and many of these organizations have a particular focus on gender politics. The political and intellectual climate in Ankara may provide its residents with greater exposure to information, politics, and practices related to gender politics compared to Istanbul, which is primarily a commercial and financial center. Participants in Ankara demonstrate higher levels of support for gender equality compared to those in Istanbul. This variation can be understood through Gramsci’s theory of hegemony, which suggests that dominant ideologies become internalized in different ways depending on socio-political contexts. In Ankara, where political and educational institutions may be more open to progressive ideologies, gender-equitable attitudes are more prevalent. In contrast, the hegemonic patriarchal norms in Istanbul may be more deeply entrenched, influencing the less progressive attitudes observed there.

##### 4.2.2.5 Education

Respondent’s higher educational level is associated with positive gender perception. One year increase in education would decrease gender inequality index score by 0.762. Educated women gain more knowledge and understanding about gender (in)equality and, as a result develop a greater awareness of gender issues. Moreover, women who have attained higher education are inclined to participate in the labor force, with a female labor force participation rate of 65.6% among those who have graduated from higher education programs (TUIK, 2021). Educated women as breadwinner positions are able to break down the patriarchal stereotypes and become less tolerant to gender hierarchy. The intersection of education, socio-economic status, and political ideology significantly shapes gender attitudes among respondents. Those with higher education levels and left-leaning political views tend to hold more progressive gender attitudes. However, even among these groups, traces of patriarchal norms persist, which can be explained by the pervasive nature of symbolic violence. Bourdieu’s framework helps us understand how patriarchal values are reinforced within social structures, making them difficult to fully challenge, even by those who recognize and oppose them. Similarly, Gramsci’s notion of hegemony highlights how dominant ideologies are maintained not through coercion but through consent, suggesting that deeper shifts in gender attitudes require more than just educational attainment.

As such, the respondents’ actual attitudes towards gender equality are not as gender-conscious as expected, and these gender inequitable attitudes vary depending on (1) religiosity, (2) political ideology, (3) age, (4) resident city and (5) education. While age and religiosity increase inequality perceptions, education, living in Ankara, and being leftist are the variables that significantly reduce inequality perceptions.

While the data reveal that participants in Ankara demonstrate more support for gender equality compared to those in Istanbul, this difference can be partially attributed to higher educational attainment levels among respondents in Ankara. Educational background, when intersected with geographical location, offers a nuanced understanding of gender attitudes. Furthermore, political ideologies and socio-economic variables interact to shape these attitudes, with left-leaning participants generally showing more gender equitable views. This intersectional approach highlights how multiple social categories—such as education, political affiliation, and geography—collectively influence gender perceptions, underscoring the complexity of the gender inequality issue within civil society. The finding that participants in Ankara show more support for gender equality compared to those in Istanbul requires a more detailed analysis of the contributing factors. One key factor is the higher educational attainment levels in Ankara, which correlate with greater awareness of gender issues. Respondents in Ankara have, on average, more years of formal education than those in Istanbul, and educational attainment is known to significantly influence attitudes toward gender equality. Thus, the higher support for gender equality in Ankara can be partially explained by this educational advantage.

Additionally, when socio-economic status is taken into account, we observe that individuals with higher incomes, regardless of location, tend to exhibit more progressive gender attitudes. This suggests that the differences in support for gender equality are not solely geographical but are also shaped by socio-economic factors. The interaction between these variables highlights the need for an intersectional approach in interpreting the data. Without accounting for how education, socio-economic status, and political ideology intersect with geographical location, the results risk oversimplification.

To further illustrate, respondents in Istanbul with higher education levels and left-leaning political views showed similar levels of support for gender equality as their counterparts in Ankara. This suggests that it is not merely the geographical location driving attitudes but the interaction of education, socio-economic status, and political ideologies that collectively shape gender perceptions.

## 5 Conclusion

This study complicates the assumption that civil society is inherently progressive. The findings reveal that gender inequitable attitudes persist within women’s NGOs, challenging the notion that these organizations are naturally aligned with feminist principles (Bourdieu, 1998). By examining the intersection of education, socio-economic status, and political ideology, this research highlights the need for internal reflection within civil society organizations. In line with Bourdieu’s theory of symbolic violence, it becomes clear that patriarchy can subtly persist even in spaces advocating for equality.

Civil society does not intrinsically have the liberating potential for plural and democratic society. It could be a site for democratic backsliding instead of democratization. In Turkish case, women NGOs has both qualitatively and quantitatively increased since 1980’s. This increase brought legal, political and institutional progress. Although the literature overemphasizes the progressive aspects of the rise of civil society, this paper uncovers new, potentially negative elements of civil society. Between November 2017 and February 2018, a survey was carried out with 735 members of women’s associations in İstanbul and Ankara. The respondents in this research do not necessarily represent their associations but they give us valuable input about women who are members of women’s association. We could expect them to be politically active and to have the lowest score in Gender Inequality Index, but the results do not support these expectations. It is revealed that members of women’s NGO’s do hold gender inequitable attitudes.

To understand this unexpected result, it is primarily important to escape from essentialism and accepts that all women organizations do not have to adhere feminist principles. Patriarchal beliefs can still exist within these organizations and threaten gender equality. In Turkish case, there are certain structural factors that curbs the agency and influence of women NGOs. Until the end of 1980’s, state has dominated over gender politics and do not give room for women to raise their voices. After the neo-liberal turn, Türkiye has achieved notable progress especially in legal and institutional terms on the way of gender equality, it is still not satisfactory. Patriarchy is even persistent within women’s organization as demonstrated by our research. The survey results offer insight into how gender and patriarchy interact with other social factors like age, religion, and political beliefs. Survey results show that older women and more religious women have lower gender equality perspective. At last, by demonstrating the persistence of patriarchy within women’s civil society organizations, the survey results give hints for the prospective researchers to study the abuse of civil society (practices & discourses) in case of Turkish women NGOs and their potential risk of deteriorating feminist movement.

Theoretically, this study challenges essentialist assumptions about women’s NGOs as inherently progressive spaces that automatically promote feminist principles. By revealing the persistence of patriarchal attitudes even within organizations that advocate for women’s rights, the study contributes to a more nuanced understanding of civil society’s role in gender politics. Drawing on frameworks such as Bourdieu’s symbolic violence and Gramsci’s hegemony theory, this research highlights how dominant ideologies can be internalized, even by those advocating for change. This has implications for scholars studying civil society, gender equality, and the complex dynamics of internalized patriarchy within advocacy organizations.

Practically, the findings of this study offer several insights for NGOs and policymakers working to promote gender equality in Türkiye. First, NGOs should implement internal training programs focused on raising gender consciousness among their members, addressing the patriarchal attitudes that persist even within women’s rights organizations. These programs should be designed to critically engage members with feminist principles and encourage reflection on how personal and institutional biases may hinder progress.

Policymakers should also collaborate more closely with women’s NGOs to incorporate grassroots insights into national gender policies. Establishing platforms where NGOs can directly influence policy development can ensure that women’s voices are represented in decision-making processes. Moreover, special attention should be paid to addressing the intersection of education and socio-economic status when developing gender equality initiatives, ensuring that efforts are inclusive and reach all demographics.

Other countries can learn from Türkiye’s experience by recognizing that legal reforms and the establishment of NGOs are not sufficient for achieving gender equality. Structural and cultural factors, such as internalized patriarchy and socio-political constraints, must also be addressed to create lasting change. The Turkish case serves as a reminder that ongoing efforts and vigilance are required to maintain and advance gender equality, even in progressive spaces.

Given the significant political and social changes in Türkiye since 2017, such as the country’s withdrawal from the Istanbul Convention and the rise of anti-LGBT rhetoric, future research should explore how these developments have affected the role and efficacy of women’s NGOs. Further studies could examine the long-term impact of these shifts on gender attitudes within civil society and whether NGOs have adapted their strategies in response to the increasingly conservative political environment.

Additionally, comparative research between urban and rural areas, or across different regions of Türkiye, would provide deeper insights into how geographical and socio-economic factors influence gender perceptions. Future research could also investigate the effectiveness of NGO-led training programs in shifting gender attitudes and explore how NGOs can navigate the challenges of internalized patriarchy to better serve their mission of promoting gender equality.

## Data Availability

The raw data supporting the conclusions of this article will be made available by the authors, without undue reservation.

## References

[ref1] Abadan-Unat N. (1978). The modernization of Turkish women. Middle East J. 32, 291–306.

[ref3] Anyanwu J. C. (2016). Accounting for gender equality in secondary school enrollment in Africa. Afr. Dev. Rev. 28, 170–191. doi: 10.1111/1467-8268.12188

[ref4] Arat Y. (1994). Toward a democratic society: the women's movement in Türkiye in the 1980s. Women's Stud. Int. Forum 17, 241–248. doi: 10.1016/0277-5395(94)90030-2

[ref5] Arat Y. (2010). Religion, politics and gender equality in Türkiye: implications of a democratic paradox? Third World Q. 31, 869–884. doi: 10.1080/01436597.2010.502712, PMID: 20857566

[ref6] Aydin H. (2015). Meşrutiyet’ten Cumhuriyet’e Türkiye’de Kadın. Curr. Res. Soc. Sci. 1:3.

[ref7] Ayhan E. (2020). Civil society organizations (CSOs) in Türkiye: History, theories and issues. Ankara: İksad Yayınevi.

[ref9001] Bourdieu P. (1998). Practical reason: On the theory of action. Stanford, CA: Stanford University Press.

[ref8] Campbell M. S. A. W. Bartkowski J. P. Acevedo G. A. Karakeci G. Favor P. N. C. Wani G. Q.. (2018). International Institute of Islamic Thought (IIIT). Am. J. Islam. Soc. Sci. 35, 25–56.

[ref9] Esim S. Cindoğlu D. (1999). Women’s organizations in1990s Türkiye: predicaments and prospects. Middle East. Stud. 35, 178–188. doi: 10.1080/00263209908701261

[ref10] Eslen-Ziya H. Kazanoğlu N. (2020). De-democratization under the new Türkiye? Challenges for women’s organizations. Mediterranean Polit. 27, 101–122. doi: 10.1080/13629395.2020.1765524

[ref11] European Parliement (2012). Gender equality in Türkiye. Available at: https://www.europarl.europa.eu/RegData/etudes/note/join/2012/462428/IPOL-FEMM_NT(2012)462428_EN.pdf (Accessed March 15, 2023).

[ref12] Fitzsimmons S. Özbilgin M. F. Thomas D. C. Nkomo S. (2023). Equality, diversity, and inclusion in international business: a review and research agenda. J. Int. Bus. Stud. 54, 1402–1422. doi: 10.1057/s41267-023-00642-x

[ref13] Frankel B. (1983). Beyond the state?: Dominant theories and socialist strategies. London: Macmillan International Higher Education.

[ref14] Freidenvall L. (2013). “Step by step – Women’s inroads to parliamentary politics” in Breaking male dominance in old democracies. eds. Dahlerup D. Leyenaar M. (Oxford: Oxford University Press), 97–123.

[ref15] Gökçi̇men S. (2008). Ülkemizde Kadınların Siyasal Hayata Katılım Mücadelesi. Yasama Dergisi 10:10.

[ref16] Goldscheider F. Goldscheider C. Rico-Gonzalez A. (2014). Gender equality in Sweden: are the religious more patriarchal? J. Fam. Issues 35, 892–908. doi: 10.1177/0192513X14522236

[ref17] Göle N. (1994). “Toward an autonomization of politics and civil society in Türkiye” in Politics in the third Turkish republic. eds. Heper M. Evin A. (Westview: Boulder).

[ref18] Habermas J. (1997). Kamusallığın Yapısal Dönüşümü. 16th Edn. İstanbul: İletişim Yayınları.

[ref19] Heinemann I. Stern A. M. (2022). Gender and far-right nationalism: historical and international dimensions. Introduction. J. Modern Eur. Hist. 20, 311–321. doi: 10.1177/16118944221110721

[ref20] Howell J. Mulligan D. (2004). Gender and civil society. İstanbul: Routledge.

[ref21] Jailobaeva K. (2007). Civil society from Liberal and communitarian perspectives. Available at: www.academia.edu.tr (Accessed March 10, 2023).

[ref22] Joseph S. (1996). Patriarchy and development in the Arab world. Gend. Dev. 4, 14–19. 12291310 10.1080/741922010

[ref23] Kane E. W. Sanchez L. (1994). Family status and criticism of gender inequality at home and at work*. Soc. Forces 72, 1079–1102. doi: 10.2307/2580293

[ref24] Kardam N. Ertürk Y. (1999). Expanding gender accountability? Women’s organizations and the state in Türkiye. Int. J. Organ. Theory Behav. 2, 167–197. doi: 10.1108/IJOTB-02-01-02-1999-B007

[ref25] Kaymaz İ. Ş. (2010). Çağdaş Uygarlığın Mihenk Taşı: Türkiye’de Kadının Toplumsal Konumu. Atatürk Yolu Dergisi 12:46. doi: 10.1501/Tite_0000000328

[ref26] Kehn A. Ruthig J. C. (2013). Perceptions of gender discrimination across six decades: the moderating roles of gender and age. Sex Roles 69, 289–296. doi: 10.1007/s11199-013-0303-2

[ref27] Keith D. J. Verge T. (2018). Nonmainstream left parties and women’s representation in Western Europe. Party Polit. 24, 397–409. doi: 10.1177/1354068816663037

[ref28] Keyman E. F. (2000). Türkiye ve radikal demokrasi: Geç-modern zamanlarda siyaset ve demokratik yönetim. İstanbul: Alfa.

[ref29] Keyman E. F. İçduygu A. (2003). Globalization, civil society and citizenship in Türkiye: actors, boundaries and discourses. Citizsh. Stud. 7, 219–234. doi: 10.1080/1362102032000065982

[ref30] Kittilson M. C. (2011). Women, parties and platforms in post-industrial democracies. Party Polit. 17, 66–92. doi: 10.1177/1354068809361012

[ref31] Klingorová K. Havlíček T. (2015). Religion and gender inequality: the status of women in the societies of world religions. Moravian Geograp. Rep. 23, 2–11. doi: 10.1515/mgr-2015-0006

[ref32] Konan B. (2011). Türk Kadınının Siyasi Hakları Kazanma Süreci. Ankara Üniversitesi Hukuk Fakültesi Dergisi 60, 157–174. doi: 10.1501/Hukfak_0000001622

[ref33] Korolczuk E. Boczkowska K. (2022). How gender became central to far-right politics. Green European Journal. Available at: https://www.greeneuropeanjournal.eu/how-gender-became-central-to-far-right-politics/ (Accessed March 1, 2023).

[ref34] Marin R. R. (2015). The constitutional status of women in Türkiye at a crossroads: reflections from comparison. Available at: https://verfassungsblog.de/constitutional-status-women-Türkiye-crossroads-reflections-comparison/ (Accessed April 15, 2023).

[ref35] Narayan-Parker D. (2005). Measuring empowerment: Cross-disciplinary perspectives. Washington, DC: World Bank Publications.

[ref36] Osborne T. (2021). Civil society, populism and liberalism. Int. J. Politics Cult. Soc. 34, 175–190. doi: 10.1007/s10767-020-09377-1, PMID: 37674595

[ref37] Özbilgin M. F. Erbil C. (2024a). “Rainbow burning to rainbow washing: how (not) to manage LGBT+ inclusion” in Genderwashing in leadership (Emerald Publishing Limited), 135–152.

[ref38] Özbilgin M. F. Erbil C. (2024b). “How (not) to manage intersectional inclusion: questioning trans exclusion” in Encyclopedia of diversity, equity, inclusion and spirituality (Leeds: Springer Nature Switzerland, Cham), 1–11.

[ref39] Öztop H. Finkel M. (2015). Women's views about gender equality on the current social policy in Türkiye. J. Int Educ. Leadership 5, 1–13.

[ref40] Savaş G. (2018). Türkiye’de Yaşayan Bireylerin Toplumsal Cinsiyet Eşit(siz)liği Algısı—Gender (In) Equality Perception of Individuals Living in Türkiye. Akdeniz Kadın Çalışmaları ve Toplumsal Cinsiyet Dergisi 1:2.

[ref41] Schechter M. G. (2016). The revival of civil society: Global and comparative perspectives. New York: Springer.

[ref42] Schnabel L. (2016). Religion and gender equality worldwide: a country-level analysis. Soc. Indic. Res. 129, 893–907. doi: 10.1007/s11205-015-1147-7

[ref43] Seçkinelgin H. (2016). Civil society between the state and society: Turkish women with Muslim headscarves? Crit. Soc. Policy 26, 748–769. doi: 10.1177/0261018306068472

[ref44] Seguino S. (2011). Help or hindrance? Religion’s impact on gender inequality in attitudes and outcomes. World Dev. 39, 1308–1321. doi: 10.1016/j.worlddev.2010.12.004, PMID: 39420400

[ref45] Sombatpoonsiri J. (2020). ‘Authoritarian civil society’: how anti-democracy activism shapes Thailand’s autocracy. J. Civ. Soc. 16, 333–350. doi: 10.1080/17448689.2020.1854940

[ref46] Tekeli S. (1992). Europe, European feminism, and women in Türkiye. Women's Stud. Int. Forum 15, 139–143. doi: 10.1016/0277-5395(92)90047-Y

[ref47] Tepe F. F. (2014). Women’s liberation in Türkiye before the 1980s: the case of Nezihe Kurtiz. J. Int. Women's Stud. 15, 299–318.

[ref48] The New Global Order (2022). Evolution of Women’s rights in Türkiye: the fall of a democracy. Available at: https://thenewglobalorder.com/report/evolution-of-womens-rights-in-Türkiye -the-fall-of-a-democracy/ (Accessed February 15, 2023).

[ref49] Tocqueville A. (2016). Amerika’da Demokrasi. İstanbul: İletişim Yayınları.

[ref50] TUIK (2021). Women in statistics, 2020. Available at: https://data.tuik.gov.tr/Bulten/Index?p=37221&dil=2 (Accessed December 15, 2022).

[ref51] Turan S. (2000). John Dewey's report of 1924 and his recommendations on the Turkish educational system revisited. Hist. Educ. 29, 543–555. doi: 10.1080/00467600050163174

[ref9003] Turkish Civil Code. (1926). Law No. 4721. Retrieved from https://www.mevzuat.gov.tr/mevzuatmetin/1.5.4721.pdf (Accessed November 10, 2023).

[ref9002] Twinning Project. (2008). Review of twinning in Turkey: Annexes to the final report. Retrieved from https://neighbourhood-enlargement.ec.europa.eu/system/files/2019-01/annexes_to_final_report_review_of_twinning_turkey.pdf (Accessed November 10, 2023).

[ref9004] UN. (2022). Gender inequality index. Retrieved from https://hdr.undp.org/data-center/thematic-composite-indices/gender-inequality-index#/indicies/GII (Accessed March 20, 2023).

[ref52] UN Women (2020). Women’s civil society organizations of the future. Available at: https://www.unwomen.org/sites/default/files/Headquarters/Attachments/Sections/Trust%20Funds/FundGenderEquality/FGE%20Brochure%20Womens%20CSOs%20of%20the%20Future_2020_web.pdf (Accessed January 15, 2023).

[ref53] UNDP (2022a). Human Development Reports. Available at: https://hdr.undp.org/data-center/human-development-index#/indicies/HDI (Accessed May 15, 2023).

[ref54] UNDP (2022b). Türkiye ‘s Gender Equality Performance. Available at: https://www.undp.org/sites/g/files/zskgke326/files/migration/tr/UNDP-TR-TÜRKIYE-GENDER-EQUALITY-PERFORMANCE-EN.pdf (Accessed May 15, 2023).

[ref55] UNFPA (2020). Gender equality. Available at: https://turkiye.unfpa.org/en/gender-equality#:~:text=Situation%20in%20T%C3%BCrkiye&text=According%20to%20the%202020%20Gender,country%20out%20of%20156%20countries (Accessed May 14, 2023).

[ref57] VanDyck C. K. (2017). Concept and definition of civil society sustainability. Center for Strategic and International Studies (CSIS), 30.

