# Corrigendum: Breaking the chains: exploring gender inequality in Türkiye through the eyes of women NGO members

**DOI:** 10.3389/fsoc.2025.1575969

**Published:** 2025-03-18

**Authors:** Gokhan Savas, Dilek Çakır

**Affiliations:** ^1^College of Arts and Sciences Department of International Studies, American University of Sharjah, Sharjah, United Arab Emirates; ^2^Political Science and Public Administration Department, Social Sciences University of Ankara, Altındağ, Türkiye

**Keywords:** gender attitudes, civil society, gender (in)equality, women rights, patriarchy

In the published article, there was an error in the Funding statement. The article did not include the funding support provided by the American University of Sharjah. The correct Funding statement appears below.

